# Spray coagulation reduces the use of hemostatic forceps for intraoperative bleeding in gastric endoscopic submucosal dissection

**DOI:** 10.1002/jgh3.70002

**Published:** 2024-07-19

**Authors:** Yumiko Ishikawa, Osamu Goto, Shun Nakagome, Tsugumi Habu, Kumiko Kirita, Eriko Koizumi, Kazutoshi Higuchi, Hiroto Noda, Takeshi Onda, Jun Omori, Naohiko Akimoto, Katsuhiko Iwakiri

**Affiliations:** ^1^ Department of Gastroenterology Nippon Medical School, Graduate School of Medicine Tokyo Japan; ^2^ Endoscopy Center Nippon Medical School Hospital Tokyo Japan

**Keywords:** endoscopic submucosal dissection, hemorrhage, hemostasis, intraoperative complications

## Abstract

**Aims:**

During intraoperative bleeding in endoscopic submucosal dissection (ESD), switching to spray coagulation may be beneficial compared with the continuous use of swift coagulation and can reduce the need for hemostatic forceps. We retrospectively assessed the effectiveness of spray modes on intraoperative bleeding during gastric ESD.

**Methods and Results:**

A total of 316 bleeding events (156 in the Swift group and 160 in the Spray group) were consecutively recorded. In the Swift group, hemostasis was performed using the swift mode with a retracted tip of the needle‐type knife, followed by the hemostatic forceps. In the Spray group, bleeding was treated in a stepwise manner: the swift mode, the spray mode, and the hemostatic forceps. All bleeding events were assigned to one of two groups by an endoscopist who retrospectively reviewed the videos. We compared the use of hemostatic forceps, the total hemostatic time, and the cumulative hemostasis rate between the two groups.

The use of hemostatic forceps was significantly lower in the Spray group than in the Swift group (32.7% vs. 13.8%, *P* < 0.001). There was no significant difference in the total hemostatic time (Swift group, 20 s.; Spray group, 16 s.; *P* = 0.42), whereas the cumulative hemostasis rate with the knife was significantly higher in the Spray group (*P* = 0.007).

**Conclusion:**

The results suggested that spray coagulation from the tip of the needle‐type knife could reduce the use of hemostatic forceps. In gastric ESD, spray coagulation may facilitate the hemostasis of intraoperative bleeding.

## Graphical abstract

Spray coagulation mode may contribute to facilitating hemostasis of intraoperative bleeding during gastric ESD. In this retrospective comparative study, the use of hemostatic forceps was significantly decreased by switching the mode from the swift coagulation to the spray coagulation when occurring intraoperative bleeding, although there was no significant difference in the total hemostatic time. Hemostasis by the spray coagulation may save the hemostatic forceps in intraoperative bleeding during gastric ESD.

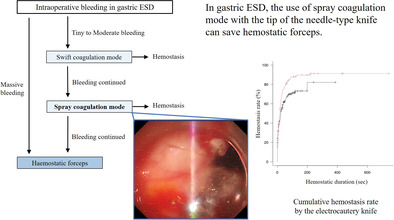

## Introduction

Endoscopic submucosal dissection (ESD) is indicated for potentially node‐negative early gastric cancers and has been widely performed as a minimally invasive curative treatment.^1^, ^2^, ^3^ In a recent aging society, ESD is also widely performed on elderly patients taking antithrombotic medications, who have a potential risk of both intraoperative and postoperative bleeding.^4^, ^5^, ^6^, ^7^ Intraoperative bleeding, which has a reported incidence of 2.9–45.1%,^8^, ^9^ can sometimes be a challenging issue in gastric ESD.^10^, ^11^, ^12^ If intraoperative bleeding is not adequately managed, several concerns may arise^13^, ^14^, ^15^, ^16^: increased intraoperative blood loss, patient instability, prolonged operative time, poor visibility of the surgical field, the use of additional hemostatic devices, thermal injury to the specimen, and increased risk of delayed perforation.

When bleeding occurs during ESD using needle‐type knives, the bleeding area is first energized to stop the bleeding with the tip of the knife.^17^ In this procedure, the subsequent use of the coagulation mode for submucosal dissection is convenient and time‐saving.^18^ However, in the swift coagulation mode, the area of energization reached is narrow and the hemostatic effect is weak; therefore, hemostasis is often not achieved quickly, and we ultimately have to resort to hemostatic forceps.^12^

Spray coagulation is a noncontact coagulation modality in which the higher voltage enables discharge through the air, which has high resistance.^19^, ^20^, ^21^ Due to the wide range of energization, hemostasis can be easily achieved compared with the swift coagulation mode, and this hemostatic capability can eliminate the need for hemostatic forceps. However, there are few studies on the hemostatic effect of different coagulation modes,^22^, ^23^ and the effectiveness of spray coagulation has been less investigated. Therefore, we retrospectively evaluated whether the spray coagulation mode led to favorable effects on intraoperative bleeding during gastric ESD.

## Methods

### Study design, ethical approval, and data collection

This study is a single‐center, retrospective study at a tertiary‐referral hospital. The study protocol was approved by the Institutional Review Board of our hospital (No. B‐2023‐715). The informed consent was obtained via an opt‐out method, and the requirement for written consent was waived due to the retrospective nature of the study.

The data were collected from the medical record and the video of the procedure. In the intraoperative bleeding management strategy, we began incorporating the spray coagulation mode into the conventional swift‐dependent hemostatic method in 2020. Therefore, except for the data from April 2020 to March 2022 as a transitional period, we consecutively collected the data on the swift mode hemostasis (the Swift group) prior to March 2020, and the data on the spray mode (the Spray group) starting from April 2022, respectively. Finally, 316 bleeding events were included in the analyses: the Swift group comprised 156 bleeding events across 25 lesions in 19 patients from December 2019 to March 2020, and the Spray group consisted of 160 bleeding events across 31 lesions in 27 patients from April 2022 to August 2022.

### ESD procedures

In the ESD procedure, we used a multi‐bending endoscope equipped with dual working channels (GIF‐2TQ260M; Olympus Corporation, Tokyo, Japan) and a transparent cap at the distal end, the 2‐mm DualKnife J electrosurgical knife (KD‐655 L; Olympus), and the Coagrasper hemostatic forceps (FD‐410LR; Olympus). An electrosurgical generator, VIO 3 (Erbe Elektromedizin, Tübingen, Germany), is used. To create the submucosal cushion, a hyaluronic acid solution (Ksmart; Olympus) diluted to five times its volume with normal saline, along with a small amount of indigocarmine and 0.05% epinephrine, was injected. Video recording was conducted in all instances. On the following day, a second‐look endoscopy was performed, and the patient resumed eating. On postoperative day 4, they were discharged. Lansoprazole 30 mg was administered intravenously intraoperatively and on the day after surgery, then switched to oral vonoprazan 20 mg on the second postoperative day and continued for one month. If symptoms of gastrointestinal bleeding, such as hematemesis or melena, were observed, emergency endoscopy was considered.

### Hemostasis for intraoperative bleeding

**Figure 1:**
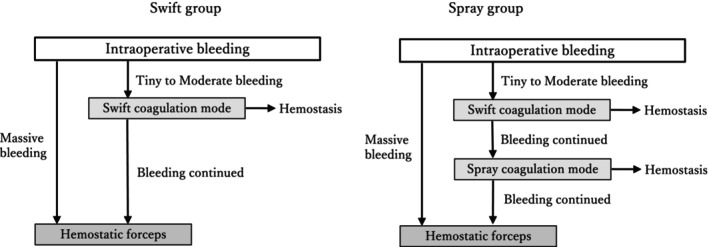
A flowchart of hemostasis. In the Swift group, intraoperative bleeding is initially treated with the swift coagulation mode, which is used for the prior submucosal dissection using the retracted tip of the electrocautery knife. If hemostasis fails after several attempts, hemostatic forceps are introduced. In the Spray group, if bleeding does not stop on the first attempt in the same manner as in the Swift group, the spray coagulation mode is applied before using the hemostatic forceps. In both groups, the hemostatic forceps are directly applied to massive bleeding.

**Figure 2:**
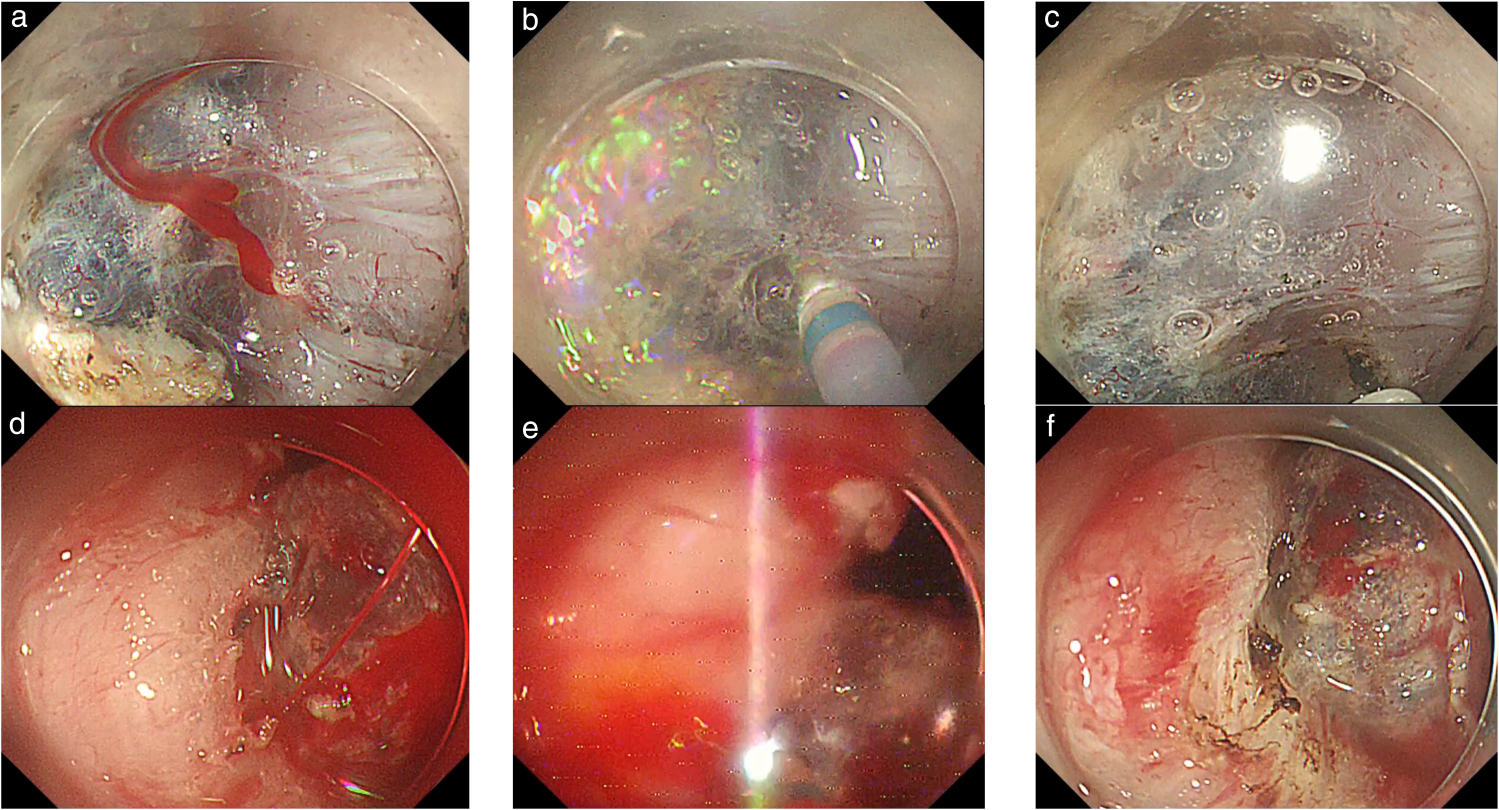
Representative images of intraoperative bleeding and hemostasis. (a) Swift group. Intraoperative bleeding occurred during the submucosal dissection. (b) A weak spark is observed at the tip of the knife. (c) Bleeding has been successfully stopped. (d) Spray group. Arterial bleeding occurred during mucosal dissection. (e) A fierce spark is observed, which is easily distinguished from the swift coagulation mode. (f) Hemostasis is achieved.

A flowchart of the strategy for hemostasis is shown in Figure 1. In the Swift group, we first attached the retracted tip of the knife to the bleeding point and cauterized it with the Swift coagulation mode of Effect 4 used for the submucosal dissection (Fig. 2a–c). If the bleeding could not be stopped after several attempts, hemostatic forceps were used to grasp the bleeding point and coagulate it with the soft coagulation mode of Effect 5. In cases of massive arterial bleeding, hemostatic forceps were used directly.

In the Spray group, swift coagulation was initially attempted using the retracted tip of the knife in a manner similar to that of the Swift group. If hemostasis is not achieved, we switch to the spray coagulation mode of Effect 4 and coagulate the bleeding point, maintaining a small distance between the tip of the knife and the target (Fig. 2d–f). If the bleeding still has not stopped, hemostatic forceps should finally be applied. Representative examples of hemostasis in both modes are shown in Videos 1 and 2.

After the removal of lesions, we performed post‐ESD coagulation (PEC) for exposed vessels on the mucosal defect.^24^ When hemostatic forceps were used for intraoperative bleeding, that was subsequently applied with the soft coagulation mode of Effect 5. In cases without the use of hemostatic forceps during ESD, we performed PEC by the retracted tip of the knife with the swift coagulation mode of Effect 4.

### Outcome measures

As the primary outcome, we evaluated the frequency of use of the hemostatic forceps. Secondary outcomes included total hemostatic time, cumulative hemostasis rate, and cumulative hemostasis rate with the scalpel. To compare the status of hemostasis between the two groups, the number of interventions per bleeding event, and the proportion of hemostatic methods were also analyzed.

One endoscopist retrospectively reviewed the videos and identified the type of coagulation mode used for hemostasis by observing the sparks generated at the tip of the knife: when the sparking was concentrated at the tip of the knife, the swift mode was used, and when the sparks dispersed to the surrounding area, the spray mode was employed. The hemostatic time was defined as the duration from the first attempt at hemostasis to the achievement of complete hemostasis. The number of energizations was defined as the number of attempts for hemostasis.

### Sample size and statistics

Due to no robust evidence published in papers, we forcibly calculated the referable sample size using previous evidence which was shown in an abstract for a domestic congress before the initiation of the analysis.^25^ In this abstract, the use of hemostatic forceps was decreased by 50% in gastric ESD by the continuous use of spray coagulation throughout the submucosal dissection. Empirically, hemostatic forceps were used in 30% of cases at our institution (the Swift group). Therefore, the use of hemostatic forceps was expected to be in 15% of the Spray group. Consequently, the required sample size was estimated at 131 events or more, considering a power of 90% and a one‐sided significance level of 0.05. Because the number of events per lesion was dependent on situation and some bleeding events might be difficult to measure the accurate hemostatic time, a certain amount of excessive number was allowed in the enrollment. All statistical analyses were performed using R for Mac (version 4.2.3).^26^ In descriptive statistics, the median and range were used for continuous variables. Chi‐square tests were used to compare categorical variables. Continuous variables were compared using the Kolmogorov–Smirnov test, with the t‐test for those following a normal distribution and the Mann–Whitney U test for those not following it. The overall cumulative hemostasis rate and the cumulative hemostasis rate with the scalpel were also analyzed using the Kaplan–Meier method. All significance levels were set at 0.05.

## Results

The clinicopathological characteristics of the Swift group and the Spray group are shown in Table 1. There were no significant differences between the two groups in terms of age, sex, and perioperative administration of antithrombotic agents as patient‐based parameters; as well as location, tumor size, specimen size, gross type, pathology, presence of ulceration, and depth as lesion‐based parameters. In the ESD outcomes of the Swift group and the Spray group, the en bloc resection rate (100% vs. 100%), the R0 resection rate (100% vs. 97%), and the rate of curative resection (86% vs. 87%) had no significant differences. Delayed bleeding occurred in three lesions in the Swift group. No significant difference was observed in the procedural time of ESD (Swift group, 45 min; Spray group, 46 min; *P* = 0.786).

**Table 1:** Clinicopathological characteristics and outcomes of endoscopic submucosal dissection (ESD).

	Swift group	Spray group	*P*‐value
Patient‐based	*n* = 19	*n* = 27	
Age (year), median (IQR)	77 (69.5–79)	74 (69.5–80.5)	0.836
Sex			1.000
Male, *n* (%)	15 (79)	21 (78)	
Female, *n* (%)	4 (21)	6 (22)	
Antithrombotic agents			0.107
None/Discontinued, *n* (%)	14 (74)	25 (93)	
Continued, *n* (%)	5 (26)	2 (7)	
Lesion‐based	*n* = 25	*n* = 31	
Location			0.467
Upper third, *n* (%)	2 (8)	6 (19)	
Middle third, *n* (%)	15 (60)	14 (45)	
Lower third, *n* (%)	6 (24)	10 (32)	
Remnant stomach, *n* (%)	2 (8)	1 (3)	
Circumference			0.524
Lesser curvature, *n* (%)	11 (44)	11 (37)	
Greater curvature, *n* (%)	3 (12)	7 (23)	
Anterior wall, *n* (%)	3 (12)	6 (20)	
Posterior wall, *n* (%)	8 (32)	6 (20)	
Tumor size (mm), median (IQR)	36 (29–44)	37 (28–45)	0.959
Specimen size (mm), median (IQR)	13 (9.5–19)	10 (8.3–18.5)	0.434
Gross type			0.422
0‐I/0‐IIa, *n* (%)	11 (44)	17 (57)	
0‐IIb/0‐IIc, *n* (%)	14 (56)	13 (43)	
Pathology			0.266
Adenoma, *n* (%)	2 (8)	0	
Differentiated type, *n* (%)	23 (92)	28 (93)	
Undifferentiated type, *n* (%)	0	1 (3)	
No tumor, *n* (%)	0	1 (3)	
Ulcerative findings			0.314
Absent, *n* (%)	22 (88)	30 (97)	
Present, *n* (%)	3 (12)	1 (3)	
Depth			0.720
pM, *n* (%)	22 (88)	26 (84)	
pSM, *n* (%)	3 (12)	5 (16)	
En bloc resection, *n* (%)	25 (100)	31 (100)	‐
R0 resection, *n* (%)	23 (100)	29 (97)	1.000
Curative resection, *n* (%)	19 (86)	26 (87)	1.000
Procedural time (min), median (IQR)	45 (34–62)	46 (32.5–61)	0.786
Adverse events			
Delayed bleeding, *n* (%)	3 (12)	0	0.083
Delayed perforation, *n* (%)	0	0	‐

IQR, interquartile range.

The hemostatic outcomes for intraoperative bleeding are shown in Table 2. The median number of bleeding episodes per lesion was six in the Swift group and five in the Spray group, with no significant difference. In terms of the primary endpoint, hemostatic forceps were used in 51 events (32.7%) in the Swift group and in 22 events (13.8%) in the Spray group, which revealed that hemostatic forceps were less frequently used in the Spray group with a significant difference (*P* < 0.001). In the patient‐based analysis, the hemostatic forceps were used in 15 patients (78.9%) of the Swift group and in nine patients (33.3%) of the Spray group (*P* = 0.005).

**Table 2:** Details of intraoperative bleeding and hemostasis

	Swift group	Spray group	*P*‐value
Lesion‐based	*n* = 25	*n* = 31	
Bleeding events per lesion (occurrences), median (IQR)	6 (2–8)	5 (3–6)	0.550
Event‐based	*n* = 156	*n* = 160	
Use of Hemostatic Forceps, *n* (%)	51 (32.7)	22 (13.8)	<0.001
Total hemostatic time (sec), median (IQR)	20 (3–56.3)	16 (3–36)	0.420
Energization per bleeding event (occurrences), median (IQR)	3 (2–6)	4 (2–7)	0.348
Hemostatic Methods			<0.001
Swift mode, *n* (%)	105 (67.3)	96 (60.0)	
Spray mode, *n* (%)	‐	42 (26.2)	
Hemostatic forceps, *n* (%)	51 (32.7)	22 (13.8)	

IQR, interquartile range.

**Figure 3:**
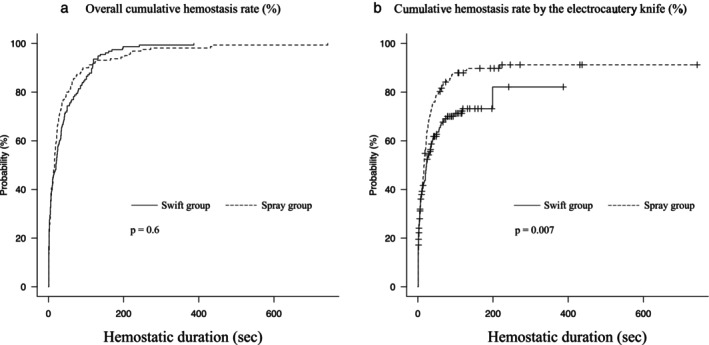
Kaplan–Meier curves of the cumulative hemostasis rates. (a) The overall hemostatic rate is not statistically different between the two groups. (b) The cumulative hemostatic rate using the knife is significantly higher in the Spray group than in the Swift group.

In the overall hemostatic time, no significant difference was observed; 20 s in the Swift group and 16 s in the Spray group (*P* = 0.42). The overall cumulative hemostatic rate was not significantly different between the two groups (Fig. 3a), while the cumulative hemostatic rate using the knife was significantly higher in the Spray group (*P* = 0.007) (Fig. 3b). There was no significant difference in the number of energizations per bleeding event (Swift group, three; Spray group, four; *P* = 0.348). Regarding the efficacy of hemostatic methods, the swift mode was effective in 67.3% of cases, while the remaining 32.7% required hemostatic forceps in the Swift group. In contrast, the swift mode, spray mode, and hemostatic forceps had effectiveness rates of 60.0%, 26.2%, and 13.8%, respectively.

## Discussion

In this retrospective study, we demonstrated that the application of the spray coagulation mode to intraoperative bleeding significantly reduced the use of hemostatic forceps during gastric ESD performed with a needle‐type knife, such as the DualKnife. Although the procedural duration, including the time for achieving hemostasis, was not affected, the spray coagulation from the retracted tip of the electrocautery knife seemed to serve as a partial replacement for hemostatic forceps in controlling intraoperative bleeding.

During ESD, Subramaniam S, et al. proposed classifying intraoperative bleeding into three categories based on the severity of the bleeding^27^: Grade 1, mild venous exudate; Grade 2, moderate nonexudative venous hemorrhage; Grade 3, severe arterial hemorrhage. In managing these bleedings, Grade 1 can be easily stopped with the tip of the knife, regardless of coagulation modes. Conversely, Grade 3 generally requires hemostatic forceps. In Figure 3b, we can interpret that Grade 1 corresponds to the initial phase where the two curves are overlapped, and Grade 3 is represented at the final phase where both curves show plateaus. In Grade 2, which appears at the intermediate phase where the curves diverge, there may be room to reduce the use of hemostatic forceps by devising hemostatic methods. This study suggests that the spray coagulation mode may contribute to stopping Grade 2 bleeding without the help of hemostatic forceps, although our way of use in the spray coagulation mode was not directly influential on the total hemostatic time as shown in Figure 3a. In fact, hemostasis was successfully achieved through the spray coagulation mode in a quarter of the bleeding cases (26.2%) in the Spray group, which was deemed to significantly contribute to the conservation of hemostatic forceps and thus lead to a decrease in the effort required for device replacement and medical expenses (15 000 yen/100 USD per the Coagrasper hemostatic forceps). In terms of the patient‐based cost, we can expect to decrease the use of hemostatic forceps in approximately 50% when using the spray coagulation mode for intraoperative bleeding.

Spray coagulation may also play a favorable role in decreasing the postoperative bleeding risk, considering three patients of bleeding in the Swift group, whereas none in the Spray group, although there was no significant difference of the bleeding rate and the number of patients were too small to discuss this matter. Furthermore, more patients in the Spray group underwent PEC with the DualKnife only, but there was no difference in the postoperative bleeding rate compared with the Swift group. This suggests that hemostatic forceps is not always necessary for PEC. The prevention ability of the spray coagulation for the delayed bleeding would be worth investigating in a future prospective study.

In Japan, the DualKnife J, which was used in this study, was introduced in 2015 as a successor to the DualKnife. Aside from the newly added flushing capability, the length of the retracted tip has been reduced from 0.3 mm in the previous DualKnife to 0.1 mm in the current DualKnife J. Consequently, minor bleeding, which was easily controlled by the tip of the previous DualKnife^28^ in the swift mode following the submucosal dissection, becomes challenging to manage with the DualKnife J. In this situation, we discovered that switching to the spray mode during bleeding facilitated the hemostasis of moderate or even some severe bleeding episodes. Due to a high‐voltage, noncontact airborne discharge, the spray coagulation can deliver effective coagulation over a broad and superficial area, which may facilitate reliable and safe hemostasis even when the bleeding point is not clearly visible. In this study, we recommend that mild bleeding can be stopped by conventional swift coagulation, while hemostatic forceps should be used for severe bleeding, and spray coagulation would be particularly useful for moderate bleeding.

No significant differences were observed in the total hemostatic time as well as the total procedural time between the two groups. In this study, spray coagulation was subsequently applied after the failure of hemostasis by the initial attempt at swift coagulation. As the study did not directly compare the hemostatic efficacy between the two coagulation modes, we believe that the effectiveness of the spray mode may not be indicative of the procedural/hemostatic time.

It can be considered that the spray coagulation mode should be used throughout the submucosal dissection. However, there is concern that the dissection surface may not be precise because the thermal effect extends over a broader area compared with the swift coagulation mode. In ESD, it is crucial to excise the entire lesion en bloc without causing thermal damage to the tumor margins. Therefore, we prefer to use the swift coagulation mode for the submucosal dissection to obtain precise and well‐defined margins of the specimen. Furthermore, we are also concerned about the tolerability of the knife in using the spray coagulation mode. That provides a high voltage, which may be a burden of the electrocautery tip. Therefore, we consider that the intensive use of spray coagulation mode should be avoided as much as possible. Indeed, there are some reports on the spray coagulation mode for submucosal dissection,^29^ and further study is needed on the effectiveness of spray coagulation in ESD. Regarding the level of the coagulation mode, the specified high‐frequency voltage for the DualKnife J is 2900 V, and the setting for the spray coagulation was set to effect level 4 in this study, which corresponds to approximately 1500 V of voltage. In fact, hemostasis is possible even at effect 1.5, and we believe that it is necessary to investigate an optimal setting for hemostasis in the future.

This study has several limitations. First, this is a single‐center, retrospective, small‐number study in patients with a wide range of bleeding risk. Second, the video was evaluated by a single endoscopist, although the discrimination between the two modes was not considered difficult. Third, the efficacy of the spray mode, which was generated by the high‐end electrosurgical unit, was not directly compared with the swift mode. Fourth, the assessment of failure in hemostasis and the timing of the switch in coagulation modes/devices were subjectively determined by each endoscopist,^30^, ^31^ and no standardized criteria were established. A well‐designed confirmatory study is needed to address these issues and to demonstrate the efficacy of the spray coagulation mode in controlling intraoperative bleeding.

In conclusion, this study suggests that the use of spray coagulation for gastric ESD‐related intraoperative bleeding improves the hemostatic rate compared with needle‐type knife coagulation and reduces the reliance on hemostatic forceps. Further prospective studies are anticipated to support these findings.

## Supporting information


**Video 1.** Hemostasis of intraoperative bleeding is sometimes challenging to achieve using the swift coagulation mode following the submucosal dissection. After several attempts with the swift coagulation, we switched to the spray coagulation mode.

**Video 2.** The hemostatic spray has sufficient potency to halt moderate bleeding, such as the spurting bleeding from a small artery.
